# Suicide in former reserve UK Armed Forces personnel, 1996–2018: A whole population cohort study

**DOI:** 10.1371/journal.pmen.0000769

**Published:** 2026-11-13

**Authors:** Jodie Westhead, Cathryn Rodway, Saied Ibrahim, Lana Bojanić, Pauline Turnbull, Louis Appleby, Andy Bacon, Harriet Dale, Kate Harrison, Nav Kapur

**Affiliations:** 1 National Confidential Inquiry into Suicide, Homicide and Safety in Mental Health (NCISH), Division of Psychology and Mental Health, School of Health Sciences, Faculty of Biology, Medicine and Health, University of Manchester, Manchester, United Kingdom; 2 Formerly of Armed Forces Team, NHS England, London, United Kingdom; 3 Westminster Centre for Research in Veterans, University of Chester, Chester, United Kingdom; 4 Ministry of Defence, Defence Statistics Health, Bristol, United Kingdom; 5 National Institute for Health and Care Research (NIHR) Greater Manchester Patient Safety Research Collaboration (GMPSRC), University of Manchester, Manchester, United Kingdom; 6 Mersey Care NHS Foundation Trust, Liverpool, United Kingdom; University of Santo Tomas College of Science, PHILIPPINES

## Abstract

“Reserve Personnel”, who serve voluntarily, comprise around a fifth of the UK Armed Forces (UKAF). Little is known about their suicide risk, or factors associated with suicide in this population. We conducted a retrospective cohort study of suicide in reserve personnel who left the UKAF between 1996 and 2018 by linking national databases. We examined the risk of suicide in this group of veterans compared to the general population and identified the factors most strongly associated with suicide after discharge. We also compared reserve veterans’ rates of and risk factors for suicide with our previous findings on regular veterans of the UKAF. The 265,941 former reserve veterans had over 4,227,131 person years at risk, and were mostly male (86%). In total, 584 (0.2%) died by suicide. Their overall rate of suicide was lower than the general population (standardised mortality ratio [95% confidence interval] 76 [70–83]), with significantly lower rates in those aged 25–29 years (age specific mortality ratios 46–87). Overall, rates were similar to age-matched regular veterans (96 [89–105]), however, risk was lower in reserve veterans aged under 25 and higher in those aged 50–59 years. Factors associated with a higher suicide risk were being male, serving in the Army, and discharge between the ages of 45–49 years. Length of service of 3–4 years and more than 5 years, and being a non-commissioned officer (NCO) were associated with a reduced risk. There was no evidence of an association with deployment. Rates of contact with specialist NHS mental health services were low (21%). Prevention should focus on ensuring reserves are aware of and have access to appropriate health, employment and social support tailored to their needs. Addressing specific stressors common in men in mid-life, such as economic adversity and isolation, may also help reduce risk.

## Introduction

The UK Armed Forces (UKAF) is comprised of full-time service personnel (regulars), Gurkhas, Volunteer Reserves and other personnel. Volunteer Reservists are those who have voluntarily accepted an annual training commitment of (normally) 27 days, as well as evening training events, and are liable to be deployed on combat operations [1]. In 2004, 20% of personnel serving in Iraq, and 12% of those serving in Afghanistan were reserves [2]. Reserves play a significant role within the UKAF and balance their civilian lives with careers across all three branches of the UKAF. Currently, reserves comprise 18% of UKAF personnel, serving across the Army, Royal Navy and Royal Air Force (RAF), with around 80% of the total reserve force in the Army [1]. The strength of the volunteer reserves (and regular forces) has decreased in recent years [1].

The experience of reservists in the UKAF differs markedly from that of regular personnel, with more time spent in the civilian world meaning less integration into military life and perhaps more similarities to the general population than the serving population [3]. It is for this reason that research examining the risk of suicide in veterans who served as reserves is sparse, with studies focusing almost exclusively on suicide risk in those who served as regulars in the UKAF [4–6], thus limiting our understanding of the factors associated with suicide in this population. Studies of US [7] and Australian [8] reservists indicate that suicide risk is lower in serving reserve personnel than in the general population, and lower in reserve veterans than in regular veterans, suggesting that reservists may not be exposed to the same experiences as regular personnel.

There is evidence that deployed reservists may have poorer mental health outcomes post deployment than their non-deployed reserve counterparts and deployed regulars [2,9–14]. An explanation for the post-deployment challenges facing reserves is that unlike regulars, they do not continue to spend time with others who were deployed, instead returning to civilian life and facing possible difficulties associated with the resumption of civilian employment, social activities, relationships, and a social support network with limited understanding of their experiences [2,9,11]. Following deployment, reservists also access civilian-focused healthcare (and welfare) that is likely to be ill-equipped to offer the appropriate support [2]. The Reserves Mental Health Programme (now part of the broader Veterans and Reserves Mental Health Programme) was developed to help tackle high rates of mental health problems amongst reserves [15] but can only be accessed by those who attribute their mental health problems to deployment [14].

Very few studies have examined the risk of suicide in reserve veterans, focusing exclusively on the health outcomes of regulars or examining reservists’ within-Service experiences, such as the mental health impact of deployment [4–6,16–20]. This is often because of a lack of available data [6], difficulties in identifying veterans with reserve service only [5], and the fact that reserves are much more heterogenous than their regular colleagues [21]. We have previously published the findings of a 23-year retrospective study of suicide in veterans who left the regular UKAF between 1996 and 2018 [4]. In this cohort, we identified 1,086 (0.2%) veterans who died by suicide. The overall risk of suicide in veterans who served as regulars was not greater than the general population, but young men and women aged under 25 years were at greater risk.

This study aimed to investigate suicide risk among UKAF reserve veterans who left service between 1996 and 2018. We: (i) examined age-specific rates and compared them to the general population; (ii) explored variation in suicide risk by demographic and service-related characteristics, including service contact; (iii) compared the characteristics of reserve veterans who died by suicide within 12 months of mental health service contact with individuals in the general population who died by suicide within 12 months of mental health contact, and (iv) compared findings with previously published results for regular veterans [4].

## Method

### Ethics statement

The following approvals were obtained for the study: Ministry of Defence Research Ethics Committee (2071/MODREC/21) on 27^th^ October 2021; the Health Research Authority Confidentiality Advisory Group provided exemption under Section 251 of the NHS Act 2006, enabling access to confidential and identifiable information without informed consent in the interest of improving care (21/CAG/0050) on 17^th^ June 2021, and the Public Benefit and Privacy Panel for Health and Social Care approved the study on 31^st^ January 2022 (2021–0290). Data were provided by the Healthcare Quality Improvement Partnership (HQIP) from the Mental Health Clinical Outcome Review Programme (MH-CORP) delivered by the National Confidential Inquiry into Suicide, Homicide and Safety in Mental Health (NCISH) (HQIP362).

### Study design

We conducted a retrospective cohort study investigating suicide risk in reserve veterans who left the UKAF between 1 January 1996 and 31 December 2018. Our cohort comprised individuals who had served exclusively as reservists (referred to as ‘reserve veterans’ herein) in any of the three branches of the UKAF (Army, Royal Navy, including the Royal Marines, and the RAF), for their entire service record (i.e., we did not include veterans who had served as both regulars and reservists). No exclusion criteria were applied to length of service and anyone who left after their first day of basic training was included, in accordance with the UK definition of a veteran [22]. The study covered England, Scotland, and Wales (data from Northern Ireland were not included due to restrictions on the sharing of identifiable data [23]). Findings for regular veterans are reported elsewhere [4].

### Procedure

We linked national databases of people who left the UKAF (collected by the Ministry of Defence in the Service Leavers Database (SLD)) and people who died by suicide (collected by the National Confidential Inquiry into Suicide, Homicide and Safety in Mental Health (NCISH)) to identify reserve veterans who died by suicide in our study period (1996–2018).

The SLD contains information on all service personnel, including reservists who have left the UKAF since the mid-1970s. An extract from the SLD was compiled by the Defence Statistics team at the Ministry of Defence on all personnel alive on the day of exit for the period 1 January 1996–31 December 2018. The extract included the name, date of birth and date of exit from Service for all personnel who left the UKAF in the study period, to enable linkage to the NCISH database. An extract of additional pseudo-anonymised variables was provided once data linkage was complete (see below).

Two databases held by the NCISH were used to identify suicide deaths in reserve veterans of the UKAF during the study period. (1) The NCISH general population suicide database contains information on all suicide deaths across the UK. This information is compiled from national mortality data provided by the Office for National Statistics (for deaths registered in England and Wales) and National Records of Scotland (for deaths registered in Scotland). Deaths assigned International Classification of Diseases, Tenth Revision (ICD-10) codes X60-X84 (intentional self-harm) or ICD-10 codes Y10-Y34 (excluding Y33.9), Y87.0, and Y87.2 (events of undetermined intent) were included in the study, as is customary for suicide research in the UK. Details on age, sex, and method of suicide were available from this general population database. Data on sex is that assigned at birth, and not self-identified. (2) The NCISH patient database includes detailed demographic and clinical information on patients who have been in contact with specialist NHS mental health services in the 12 months prior to their death. This information is collected via a questionnaire completed by the senior professional who worked with the patient in a clinical context [24].

### Data linkage

Data linkage occurred in two stages. Firstly, the Ministry of Defence’s SLD and NCISH’s general population suicide database were linked using last name, first name(s) (where available), and date of birth. More than three-quarters (n = 453, 77%) of the 584 individuals we linked between these two databases (i.e., were identified as ex-reservists who died by suicide), were matched exactly using these three variables. A further 16% (n = 91) were matched using last name, initials and date of birth. The remaining 7% (n = 40) were manually determined as suicide deaths of reserve veterans having been matched on last name and date of birth and checked and discussed by the research team (SI, CR, JW, LB). Once the first stage of data linkage was complete, the following pseudo-anonymised variables were obtained from the SLD using a unique identifier – age, sex, ethnicity (although these data were later excluded due to a large proportion of missing data (92%)), marital status, rank, training indicator, date of entry, length of service, deployment on combat operations, and date and type of discharge, and linked to the NCISH patient database.

### Statistical analysis

We used STATA 16.1 software for Windows to conduct all statistical analysis. To compare reserve veterans suicide rates to the UK general population and the regular veterans’ population, we calculated age-specific mortality ratios in 5-year age bands (see Table 1, we collapsed the oldest age-bands into one age category (65–77 years) due to small numbers) and overall standardised mortality ratios (SMRs). Age-specific suicide rates in the UK general population for the study period (1996–2018) were calculated using the number of suicides as the numerator (from the NCISH general population suicide database) and ONS annual mid-year population estimates as the denominator [25]. Age-specific suicide rates for the regular veterans’ population were estimated from our previous study [4] and used to calculate age-specific mortality ratios. A locally weighted scatterplot smoothing (LOWESS) curve was used to examine the relationship between suicide rates in reserve veterans and their age at death.

**Table 1:** Risk factors associated with suicide in reserve veterans according to different demographic and service-related characteristics.

Variable	Categories	N (at risk)^a^	Number dying by suicide	HR^b^ (95% CI)	*p*-Value^c^
**Age (years) at service exit**	16-19	16,539	24 (0.1%)	Base	0.012
	20-24	54,229	96 (0.2%)	1.26 (0.79-2.03)	
	25-29	31,981	69 (0.2%)	1.35 (0.80-2.26)	
	30-34	26,850	68 (0.3%)	1.52 (0.88-2.62)	
	35-39	17,875	46 (0.3%)	1.66 (0.91-3.05)	
	40-44	11,292	19 (0.2%)	1.25 (0.60-2.59)	
	45-49	77,571	235 (0.3%)	2.04 (1.09-3.80)	
	50-54	5,618	6 (0.1%)	1.06 (0.38-2.97)	
	55+	23,986	21 (0.1%)	0.91 (0.40-2.06)	
**Sex**	Male	228,537	557 (0.2%)	Base	<0.001
	Female	37,404	27 (0.1%)	0.30 (0.20-0.44)	
**Service**	Army	243,369	567 (0.2%)	Base	0.004
	Navy	7,386	5 (0.1%)	0.75 (0.31-1.83)	
	RAF	15,186	12 (0.1%)	0.40 (0.23-0.72)	
**Rank**	Non-officer rank**^d^**	150,996	371 (0.2%)	Base	0.03
	Commissioned Officer	20,138	20 (0.1%)	0.66 (0.42-1.03)	
	NCO	87,315	183 (0.2%)	0.81 (0.66-0.98)	
**Marital status**	Single	41,348	35 (0.1%)	Base	0.11
	Married	12,001	9 (0.1%)	0.74 (0.35-1.56)	
	Divorce/separated	742	< 5 (<0.7%)	4.43 (1.36 -14.50)	
**Training^e^**	Trained	23,488	18 (0.1%)	Base	0.30
	Untrained	32,982	29 (0.1%)	1.01 (0.56 – 1.83)	
	Unknown	209,471	537 (0.3%)	1.29 (0.79 – 2.07)	
**Type of discharge**	Voluntary (normal, own request)	159,996	406 (0.3%)	Base	0.33
	Involuntary (disciplinary, medical, administrative)	64,523	140 (0.2%)	1.11 (0.91-1.36)	
**Deployed on combat operation^f^**	No	249,566	556 (0.2%)	Base	0.80
	Yes	16,375	28 (0.2%)	0.95 (0.65-1.39)	
**Length of service (years)**	<1	30,445	75 (0.2%)	0.96 (0.66-1.38)	
	1 to <2	33,857	66 (0.2%)	0.78 (0.53-1.14)	
	2 to <3	25,282	52 (0.2%)	0.81 (0.5-1.30)	
	3 to <4	16,938	25 (0.1%)	0.58 (0.36-0.93)	
	4 to <5	9,634	19 (0.2%)	0.78 (0.47-1.30)	
	5 to <10	30,099	51 (0.2%)	0.59 (0.41-0.84)	
	10 to <15	13,424	21 (0.2%)	0.56 (0.35-0.90)	
	15+	106,262	275 (0.3%)	Base	0.01

^a^Percentages in brackets expressed as a proportion of valid cases. The number of valid cases may vary between variables (risk groups) due to missing data;

^b^Unadjusted HRs calculated using Cox’s proportional hazards modelling. Men and women both included. Total number at risk 265,941, total number of suicide deaths 584;

^c^p-Values calculated using log-rank test

^d^Refers to individuals with the rank of able mate, marine, private, aircraftman, lance corporal, senior aircraftman;

^e^Refers to individuals who have completed phase 1 (basic) and phase 2 (skill-based) training;

^f^Includes deployment to Falklands Campaign (1982), Gulf-1 (1990–1991), Iraq (2003–2011), and Afghanistan (2002–2014).

CI = confidence interval; HR = hazard ratio; NCO = Non-commissioned officer.

We investigated which risk factors were most associated with suicide after discharge from the UKAF by generating hazard ratios (HRs) and 95% confidence intervals (CIs) using Cox’s proportional hazards models, with age at death as the failure time and the end of the study period (31 December 2018) as the censor time. We focussed on univariable analyses with unadjusted HRs but also examined a multivariable model by including statistically significant risk factors and estimating adjusted HRs. In survival models, a bias could occur where we assess a period of time during which the outcome of interest cannot possibly happen [26]. In our study, this may occur when the analysis includes a period during which a person is not yet considered a ‘veteran’ (i.e., they are still serving and have not left or not yet joined the armed forces). To account for any immortal time bias, we included age as a time-varying covariate in the models and Kaplan-Meier survival curves were created and presented for significant characteristics (age at service exit, sex, Service type, and length of service). Our survival analysis was conducted using the STSET command in STATA and crude suicide rates were calculated by time since leaving the UKAF with person-years at risk as the denominator using the STPTIME command.

The NCISH patient suicide database was used to determine the rate of contact with specialist NHS mental health services in the 12 months prior to death. We used a matched case-control design to compare the characteristics of reserve veterans who had died by suicide within 12 months of NHS mental health service contact (i.e., cases) with those who had service contact in the general population who died by suicide (i.e., controls). Up to five controls were selected per case matched on age, sex, and year of death. Differences were explored using a conditional logistic regression analysis and presented using conditional odds ratios (ORs) and 95% CIs. The resulting 611 controls had no record of ever serving in the UKAF. Five controls (general population) per case (reserve veteran) gave us 80% power to examine relative risks of 2.5 and above. This is assuming a prevalence of a risk factor of 30% in the general population control group and α = 0.05.

## Results

The overall cohort of veterans who left any of the three branches of the UKAF between 1^st^ January 1996 and 31^st^ December 2018 was 723,989. There were 458,048 (63%) veterans who had previously served in the regular forces and 265,941 (38%) exclusively in the reserve forces. The remainder of this paper relates primarily to individuals who had served exclusively in the reserve forces (‘reserve veterans’), although there are some comparisons with our previously published findings on veterans who had served as regulars (‘regular veterans’) [4].

Of the 265,941 reserve veterans who had left the UKAF, the median length of follow-up (interquartile range (IQR)) was 16.7 years (12.7-19.8 years) and they had accumulated a total of 4,227,131 person years at risk. Table 1 shows the characteristics of the reserve veterans in our cohort. There were 228,537 (86%) men and 37,404 (14%) women. This reflects the gender composition of serving reserve forces [26]. The median age (IQR) of the reserve veteran cohort at last discharge from the UKAF was 35 years (24–45 years). Most (29%) were aged 45–49 years at Service exit, and the majority (92%) had served in the Army. Two percent had a medical reason for discharge. Compared to regular veterans, there were proportionally more women in our reservist cohort (14% v. 9%), reserve veterans were older at last discharge from the UKAF (35 years v. 26 years) and more had served in the Army (92% v. 62%).

### Characteristics of reserve veterans who died by suicide

Over the 23-year study period, we identified 584 (0.2%) reserve veterans who died by suicide after leaving the UKAF by linking NCISH and MoD databases. Most (557, 95%) were men; 27 (5%) were women (Table 1). Their median age (IQR) at discharge was 38 years (26–45 years); their median age (IQR) at death was 46 years (35–53 years). There were few (32, 5%) reserve veterans aged under 25 years who died by suicide, 39% (n = 230) were aged between 25 and 44 years, and 55% (n = 322) were 45 years or older. Most (97%) had served in the Army. The most common methods of suicide were hanging or strangulation (n = 318, 55%) and self-poisoning (n = 99, 17%). Firearm deaths were rare (n = 20, 3%). During the period 1996–2018, hanging or strangulation and firearm deaths were more common in reserve veterans than in the general population (hanging/strangulation (56% vs. 44%; p < 0.001); firearms (3% vs. 2%; p = 0.016)); self-poisoning was less common (17% vs. 24%; p < 0.001). Of the 584 reserve veterans who died by suicide, 193 (33%) were early service leavers (with less than 3 years of service). Reserve veterans who died by suicide after leaving the UKAF were older than regular veterans at both discharge (38 years v. 23 years) and death (46 years v. 32 years). Overall, a greater proportion of reserve veterans were aged over 45 years compared to regular veterans (55% v. 19%).

### Rate of suicide

In Table 2, we show the age-specific mortality ratio and the SMR for male reserve veterans only compared to (a) the general population and (b) male regular veterans. We do not show the age-specific mortality ratio and the SMR for female reserve veterans due to small numbers in any one age category.

**Table 2:** Numbers, crude rates per 100,000 person years and age-specific rate ratios for suicide in male reserve veterans who left the UKAF, 1996-2018.

Age at death	Number of suicide deaths	Crude rate	General population as reference population		Regular veterans as reference population	
			Age-specific RR^a^ (95% CI)	SMR (95% CI)^b^	Age-specific RR^a^ (95% CI)	SMR (95% CI)^b^
**18-24**	27	13.0	81.9 (56.1-119.4)	–	46.9 (32.2-68.4)	–
**25-29**	39	12.7	63.5 (46.4-86.9)	–	57.2 (41.8-78.3)	–
**30-34**	63	18.1	81.1 (63.3-103.8)	–	78.9 (61.6-101.0)	–
**35-39**	60	17.5	76.3 (59.2-98.3)	–	95.2 (73.9-122.6)	–
**40-44**	52	18.3	76.3 (58.1-100.1)	–	101.4 (77.3-133.1)	–
**45-49**	102	18.0	76.9 (63.4-93.4)	–	115.4 (95.1-140.2)	–
**50-54**	101	20.3	96.4 (79.3-117.2)	–	128.8 (105.9-156.5)	–
**55-59**	68	13.0	69.8 (55.0-88.5)	–	147.9 (116.6-187.6)	–
**60-64**	33	9.7	65.6 (46.7-92.3)	–	105.7 (75.1-148.7)	–
**65-77^c^**	12	5.1	40.6 (23.1-71.5)	–	82.2 (46.7-144.7)	–
**18-77^c^**	557	15.2	–	**75.9 (69.9-82.5)**	–	**96.3 (88.6-104.6)**

^a^Age-specific RR and SMR estimated using suicide rates in the general population and the regular veteran population as reference. Age-specific RR and SMR are expressed so that a ratio under 100 indicates reduced risk and a ratio above 100 indicates an increased risk of suicide.

^b^A SMR indicates the overall risk of this cohort compared to the general population.

^c^Age category up to 77 because no suicide deaths in male reserve veterans occurred in older age groups.

CI = confidence interval; RR = rate ratio; SMR = standardised mortality ratio; UKAF = UK Armed F

(a) Compared with the general population

Overall, the SMR and 95% CIs suggested the male reserve veteran suicide rate was lower than the rate in the age-matched general population (SMR [95% CI] 76 [70–83]). However, the age-specific mortality ratios and 95% CIs suggested the risk of suicide was significantly lower specifically for men aged 25–29 years, 35–49 years, and over 55 years compared to age-matched groups in the general population. Overall, there appeared to be a slight increase in the suicide rate in middle-aged reserve veterans and a lower rate in the younger and older age-groups (Fig 1). The suicide rate fell from 20.3 per 100,000 person years at ages 50–54 to 5.1 at ages 65–77 years (Table 2, Fig 1).

**Fig 1:**
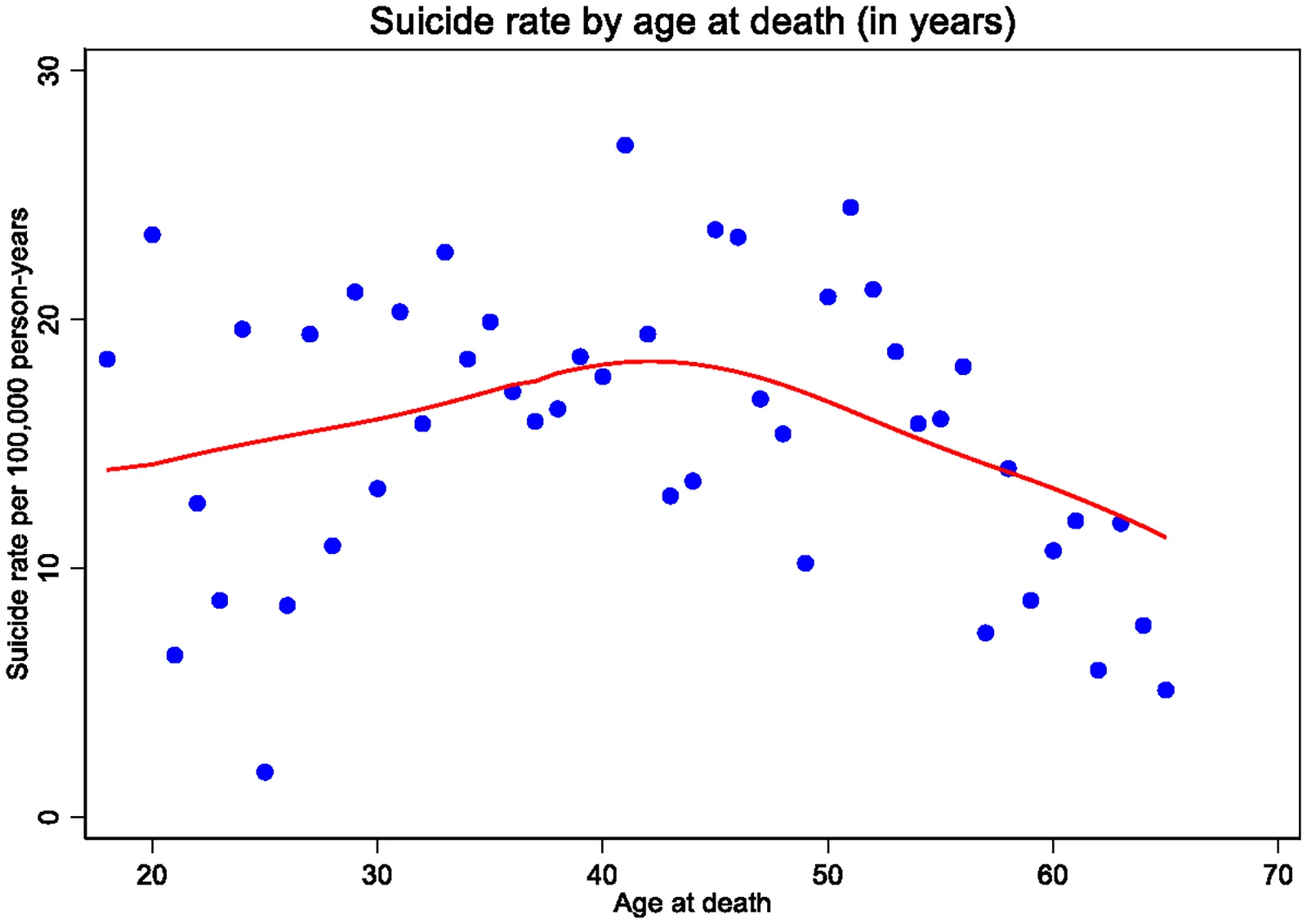
Suicide rates in male reserve veterans by age at death (in single years), with locally weighted scatterplot smoothing (LOWESS) curve.

Suicide rates across Service branches indicated a lower SMR for men who had served in the Army reserves (SMR [95% CI] 78 [72–85]). This is likely reflective of the large proportion of Army reserves who died by suicide (n = 567, 97%) compared to the Royal Navy and RAF (n = 17, 3%). We do not show the breakdown of age-specific data in female reserve veterans due to small numbers and findings relating to female reserve veterans should be interpreted with caution. The overall SMR for female reserve veterans who left the UKAF suggested their risk of suicide was not greater than the risk of suicide in the general population (SMR [95% CI] 83 [57–121]). The small number of women in the cohort of reservists who died by suicide (n = 27) means the 95% CIs were wide and did not reach statistical significance. However, unlike male reserve veterans, the findings indicate an increased risk of suicide in the youngest female age-group (18–24 years; age-specific mortality ratios [95% CI] 174 [73–418]) and in women aged 35–39 years (age-specific mortality ratios [95% CI] 120 [60–240]) compared to the same age-groups in the general population, although these findings should be interpreted with caution due to small numbers.

(b) Compared with regular veterans

A comparison of respective SMRs suggest the overall risk of suicide was very slightly (but not statistically significantly) lower in reserve veterans compared to age-matched regular veterans (SMR [95% CI] 96 [87–105]). However, the risk of suicide for the two youngest reserve veteran age-groups (18–24 and 25–29 years) was almost two times lower than age-matched regular veterans. Conversely, for men aged 50–59 years, the age-specific mortality ratios suggested the risk of suicide was higher in reserve veterans compared with age-matched regular veterans.

### Timing of suicide

Fig 2 shows the rate of suicide in male veteran reserves by time elapsed since discharge from the UKAF. This is presented for men only because of the small number of women who died by suicide in each individual year. Despite year-on-year variation, the rate of suicide appeared to be significantly higher in the second year since discharge (suicide rate [95% CI] 22.7 [17.2-29.8]) compared to the overall suicide rate in reserve veterans (15.2 per 100,000 person years)).

**Fig 2:**
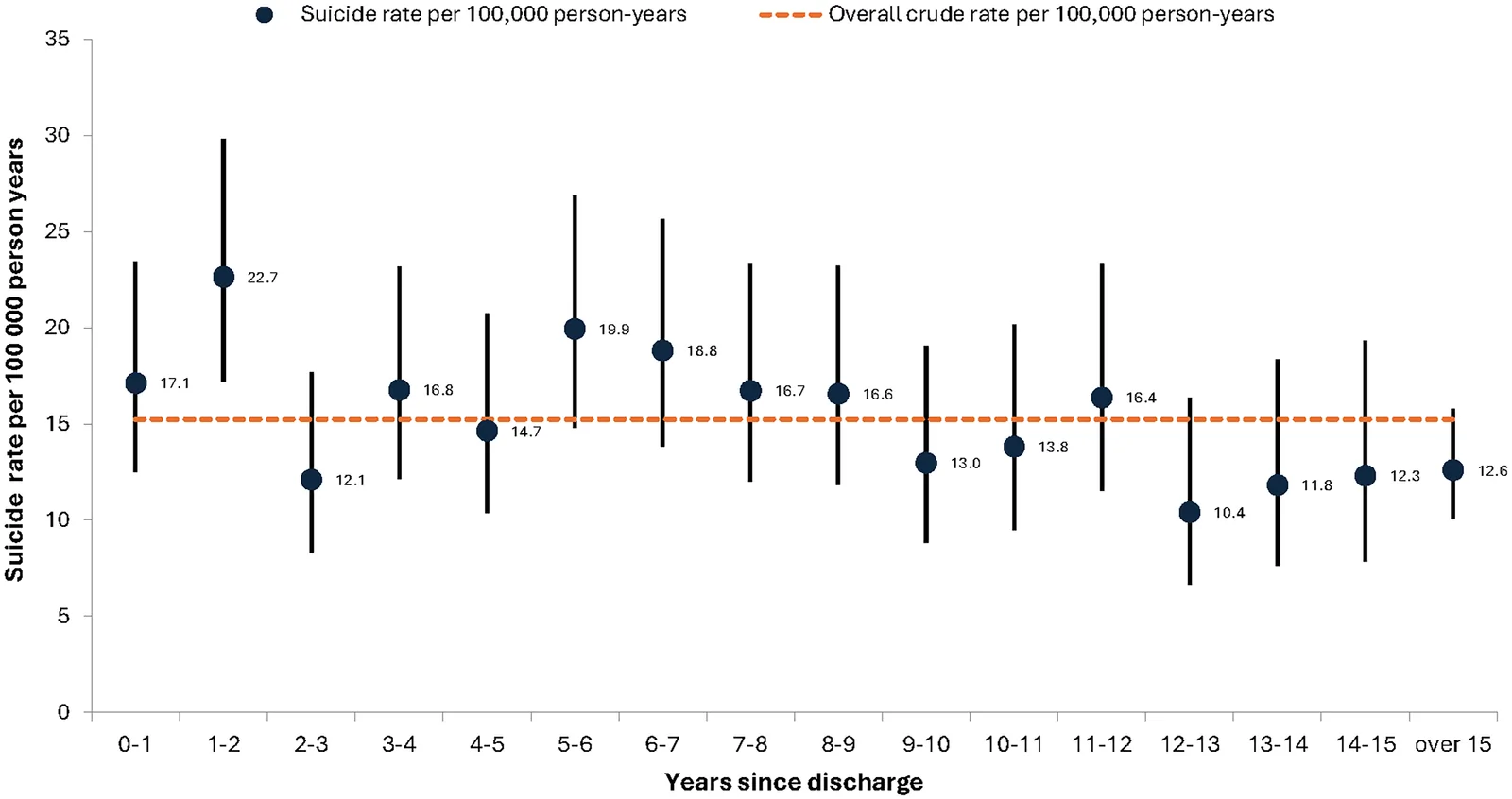
Rate of suicide in male reserve veterans by time elapsed since discharge.

### Factors associated with suicide in reserve veterans according to different characteristics

In our cohort of reserve veterans, higher suicide risk was associated with being male and being discharged from the UKAF between the ages 45–49 years (Table 1). After adjusting for multiple risk factors, veterans being discharged between the ages 35–39 years were also at a higher risk compared to younger reserve veterans (see S1 Table). Deployment was not associated with higher suicide risk. Having served for 3–4 years or for more than 5 years, being a non-commissioned officer (NCO) and serving in the RAF compared to the Army were associated with lower suicide risk. The supplementary data (S1 Fig) presents Kaplan-Meier survival estimates of reserve veterans who died by suicide according to selected risk factors (i.e., those risk factors most associated with suicide by veterans after discharge; sex, age at discharge, length of service and type of Service). The curves correspond with the HRs shown in Table 1.

Being over 45 years old at service exit, serving in the Army, being an NCO, and serving for over 15 years were more common in reserve veterans compared to regular veterans who died by suicide, although their suicide risk was no higher than the general population. Those who served in the Army being at greater risk may be driven by the larger number of suicide deaths in this branch and reflect the large percentage of the reserve force that serve in the Army. Reserve veterans were less likely to have been deployed on combat operations or to have left service involuntarily than their regular veteran counterparts (S2 Table).

### Rates and characteristics of reserve veterans in contact with mental health services prior to suicide

One hundred and twenty-three (21%, 95% CI 18–25) of the 584 people who died by suicide after leaving the reserves forces, had been in contact with NHS mental health services in the 12 months prior to their death. The proportion of people who had left the UKAF and had contact with mental health services prior to suicide was lowest in the youngest and oldest age groups, although numbers were small (4% in those aged under 25 years; 1% in those aged over 65 years).

The characteristics of reserve veterans who died by suicide within 12 months of contact with NHS mental health services (cases) are shown in Table 3. They are compared with people matched by age, sex, and year of death, who also died by suicide within 12 months of contact with NHS mental health services but had never served in the UKAF (controls). There were few differences between cases and controls. Reserve veterans who died by suicide were more likely than their respective controls to have evidence of a post-traumatic stress disorder (PTSD) (including a primary, secondary, tertiary, or quaternary diagnosis), although numbers were small and 95% CIs wide, a short duration of illness (of less than 12 months) and to have had their first contact with NHS mental health services within the 12 months prior to death. They were less likely to be unmarried or unemployed at the time of death and to have a diagnosis of drug dependence or misuse, although numbers for a diagnosis of drug dependence or misuse were small and should be interpreted with caution.

**Table 3:** Characteristics of reserve veterans of the UKAF and who had contact with mental health services in the 12 months prior to suicide and matched controls who had never served in the UKAF.

Risk group	Variable	Cases (n = 123)^a^	Controls (n = 611)^a^	*p*-Value	OR (95% CI)
**Demographic features**	Unmarried	80 (67%)	455 (77%)	0.03	0.63 (0.41-0.96)
	Unemployed	37 (32%)	303 (53%)	<0.01	0.41 (0.26-0.64)
	Long-term sick	18 (16%)	112 (19%)	0.37	0.77 (0.44-1.34)
	Living alone	51 (44%)	312 (53%)	0.06	0.67 (0.44-1.01)
	Homeless	<5	13 (2%)	0.74	0.80 (0.18-3.57)
**Service characteristics**	In-patient	11 (9%)	53 (9%)	0.92	1.04 (0.53-2.06)
	Recent (< 3 months) discharge	24 (21%)	97 (17%)	0.32	1.39 (0.82-2.34)
	Under CR/HT services	15 (15%)	72 (14%)	0.75	1.09 (0.59-2.01)
	Missed last contact	30 (28%)	148 (27%)	0.90	0.95 (0.60-1.52)
	Non-adherent with medication	15 (13%)	78 (14%)	0.79	0.91 (0.51-1.64)
**Clinical characteristics**	Schizophrenia & other delusional disorders	21 (17%)	131 (22%)	0.25	0.74 (0.44-1.24)
	Affective disorders (depression or bipolar disorder)	48 (39%)	250 (42%)	0.64	0.90 (0.60-1.35)
	Alcohol dependence/misuse	12 (10%)	51 (9%)	0.63	1.17 (0.60-2.27)
	Drug dependence/misuse	<5	47 (8%)	0.01	0.20 (0.05-0.82)
	Personality disorder	9 (7%)	47 (8%)	0.86	0.94 (0.44-1.98)
	Any PTSD diagnosis^b^	<5	<5	0.01	7.2 (1.20-43.03)
	Any secondary diagnosis	66 (55%)	320 (53%)	0.81	1.06 (0.71-1.57)
**Behavioural characteristics**	History of illness < 12 months	30 (27%)	98 (17%)	0.02	1.76 (1.08-2.87)
	History of self-harm	79 (66%)	389 (65%)	0.83	1.06 (0.69-1.63)
	History of violence	36 (31%)	163 (29%)	0.58	1.14 (0.73-1.79)
	History of alcohol misuse	70 (59%)	327 (56%)	0.46	1.21 (0.80-1.83)
	History of drug misuse	40 (35%)	261 (44%)	0.06	0.60 (0.38-0.95)
**Contact with services**	First contact with MHS < 12 months	39 (39%)	126 (25%)	<0.01	2.02 (1.27-3.22)
	Last contact < 7 days	57 (48%)	286 (48%)	0.95	1.02 (0.68-1.54)
	Long-term risk: low or none	56 (54%)	304 (58%)	0.40	0.78 (0.51-1.21)
	Short-term risk: low or none	91 (82%)	465 (84%)	0.64	0.89 (0.51-1.53)

^a^Percentages in brackets expressed as a proportion of valid cases. The number of valid cases may vary between variables (risk groups) due to missing data;

^b^Includes a primary, secondary, tertiary, or quaternary diagnosis of PTSD. CI = Confidence interval; CR/HT = Crisis Resolution/Home Treatment; OR = Odds ratio;

PTSD = Post-traumatic stress disorder; MHS = Mental Health Service; UKAF = UK Armed Forces

When comparing reserve and regular veterans who died by suicide within 12 months of contact with NHS mental health services, we found few differences. Reserve veterans were more likely than regular veterans to be on long-term sick leave and were less likely to be unemployed or have a history of drug misuse (S3 Table).

## Discussion

### Main findings

Between 1996 and 2018, 265,941 veterans left the reserve armed forces; 584 (0.2%) died by suicide. We found the risk of suicide in male reserve veterans was lower than men in the general population. The risk may have been at its highest in the two years following discharge, with year-on-year variation in the years thereafter. Suicide risk was greatest in men, those who served in the Army, and in those who left service between the ages of 45–49 years. The increased risk among men and Army personnel is consistent with patterns reported in the serving Armed Forces population [27]. Deployment on combat operations was not associated with an increased risk of suicide in our sample of reserve veterans, however, this finding should be interpreted with caution due to the small number of reserve veterans who were deployed, which may have limited statistical power.

Compared to regular veterans, the risk of suicide in reserve veterans was similar overall (SMR 96 [87–105]), although there were differences according to age. We previously reported a higher risk of suicide in young regular veterans aged under 25, but no elevated risk was apparent in young reservists of the same age. However, we did find higher rates of suicide in middle-aged (50–59 years) reserve veterans compared to regular veterans. This may reflect pre-service exposures and risk in young people who join the regular UKAF but perhaps a protective effect of service for regulars in midlife.

The rate of contact with specialist mental health services in the 12 months prior to death was comparatively low (21%) compared to the general population (26%) [28] and lower than the rate of contact observed in veterans of the regular UKAF (25%) [4]. Although firearm-related deaths were rare in reserve veterans (3%), and findings should therefore be treated with caution, they were higher than in the general population (2%) [29]. This is a very small difference in absolute risk but could represent an increase in relative risk reflecting greater familiarity and access to firearms among reserve veterans. What is perhaps more striking is the much lower rate of firearm suicide in our study compared to rates found among veterans who died by suicide in the US [30], likely reflecting greater accessibility and fewer restrictions.

### Strengths and limitations

To our knowledge this is the first and largest whole population study to systematically examine suicide risk in a cohort of individuals who had served as reserves and had been discharged from the UKAF, with over 265,000 reserve veterans and up to 23 years follow-up. There are very few studies that have focused on the health outcomes of reserve veterans in this way, and it has allowed us to identify key characteristics for suicide prevention; being male, serving in the Army and being discharged between the ages of 45–49 years. Our findings must be considered in the context of the following methodological limitations.

Information on reserve veterans was obtained from administrative databases, and we were limited to the information within them. For some variables, including ethnicity and marital status, there was a large proportion of missing data (~80–90%) and we are therefore unable to comment on the role of ethnicity or marital status in this population. The proportion of missing data also limits the generalisability of the findings and limits any interpretation of the data on specific variables such as marital status and ethnicity.

Our analysis of clinical characteristics was only for reserve veterans identified as having been in recent (less than 12 month) contact with specialist NHS mental health services. We were unable to identify reserve veterans who may have sought support from their GP, the Emergency Department or from other health, social care or voluntarily services. Our findings may therefore underestimate healthcare contact prior to suicide. We were also unable to explore factors such as employment, living circumstances, bereavement, and self-harm that may have contributed to suicide risk. These factors have, however, been explored in our earlier in-depth examination of a sample of veteran (regular and reserve) suicide deaths using coroner records [31].

The NCISH general population suicide database may include some people who were serving in the UKAF at the time of their death. We were unable to exclude these deaths as we did not have the data to identify them. However, their number is likely to be small [27], approximately 0.8% of all suicide deaths in the general population [30]. Therefore, this is unlikely to affect the rate of suicide compared to the general population that we report in this study.

Due to a lack of published age-specific suicide rates for serving reservists, we were unable to calculate SMRs to compare rates of suicide in reserve veterans who had left the UKAF with reserves who were serving in the UKAF between 1996 and 2018. Our previous study indicated suicide risk was over two times higher in regular veterans compared with serving personnel [4], but we are unable to comment on comparative risks in our reserve cohort.

We also did not aim to compare reserve veterans with nonveterans and have therefore not included a matched comparison group of nonveterans in our analysis. Previous research suggests reserves, by the very nature of their role, share more similar characteristics to the general than the military population [2].

Conducting research that links sensitive data between multiple sources is complex, requiring the approval of several regulatory bodies that function independently. The process of linking, analysing and interpreting such data also necessitates sufficient time to ensure validity and real-world application. These factors, alongside delays in legal processes (i.e., the time lag between an unexpected death and it receiving a coroner conclusion at inquest), contribute to a time intensive project. Whilst the cohort reported on in this paper may now be historical, the number of additional registered suicide deaths since 2018 are likely to be small and therefore unlikely to significantly impact the findings of this paper.

### Interpretation and implications

What do our findings tell us and how might we interpret (1) the lower risk of suicide in reserve veterans compared to the general population and (2) the differences according to age when compared to regular veterans? One possible explanation is that reserves could be healthier than the general population – an example of the “healthy worker effect” [32]. Reserve veterans may maintain stronger ties to civilian life, such as continued civilian employment and social support, which could ease adjustment following deployment or discharge. In addition, a role in the UKAF may provide opportunities not always available in civilian life – overseas travel, adventure, enhancing training and skills, and comradeship. However, the elevated risk of suicide found following the second year of discharge could highlight delayed difficulties in transiting fully from serving as a reserve to civilian life.

Reserves being healthier may also be relevant when interpreting the lower rate of suicide in the youngest age groups when compared to regular veterans. It is possible that young regular veterans experienced more pre-service vulnerabilities. The elevated risk of suicide for reserves aged 50–59 years when compared to regulars highlights the importance of considering factors associated with suicide in men in mid-life and the pressure faced by reserves juggling the demands of civilian life with their military duties.

The median age at death in our sample was 46 years, and men who left the UKAF in their forties appeared to be at an elevated risk of suicide compared to regular veterans of the same age. The highest suicide rates in the general population are in men aged 45–49 years [33]. Our earlier work has found many of the characteristics and stresses facing veterans who died by suicide are consistent with established risk factors for suicide in men in mid-life [34] such as isolation, alcohol and drug misuse, and self-harm. Prevention strategies should seek to address these by supporting reserve veterans with the dual challenges of both service and civilian life.

One in 5 reserve veterans were in contact with NHS specialist mental health services. Those who had left the UKAF were more likely than those who had not served to have a short duration of illness and to have had their first contact with mental health services in the 12 months prior to death, signalling the need for quick intervention for early onset difficulties. These findings could reflect difficulties in accessing mental health support either from regular NHS services or from services specifically for veterans, with reservists “falling through the gap”. The Veterans and Reserves Mental Health programme [15] is only accessible to reservists who attribute mental ill-health to their experiences of deployment and excludes reserve veterans who were not deployed or who do not attribute their mental health difficulties to deployment. Access typically relies on a clinical referral and is only available at a limited number of sites across the UK. Civilian healthcare, on the other hand, may also be limited in the support it can provide to reserve veterans with services encountering difficulties in assessing the treatment needs of individuals who have fulfilled a dual civilian/military role. Healthcare options for veterans have improved since 2010, with the development of NHS veteran-specific services such as Op-COURAGE. Op-COURAGE provides mental health support to serving personnel due to leave the military, reservists and Armed Forces veterans residing in England [35] and can liaise with other NHS services or charities on a veteran’s behalf [36]. However, some reservists may not identify as veterans or there may be other factors preventing them from seeking mental health support, such as stigma or a perception that healthcare services and charities would not understand their needs, or that they would not benefit from accessing support.

Our findings suggest mental health provision for reserves should focus on providing more continuity between defence and NHS medical services and systems, being more inclusive and accessible, and ensuring support is available to all reserves regardless of their experiences of deployment. This is particularly relevant given plans to increase the number of reserve personnel [3]. Reserve veterans should be signposted to how and where they can access support during transition out of and post-service. This awareness is crucial in the years immediately after discharge where the risk of suicide appears to be slightly elevated and where transition out of service and the adaption to solely civilian life may coincide with other factors in mid-life (e.g., economic adversity, isolation, alcohol and/or drug misuse) that could exacerbate suicide risk.

## Supporting information

S1 Table Multivariable analyses with adjusted hazard ratios on significant risk factors associated with suicide in reserve veterans. (PDF)

S1 Fig Kaplan-Meier survival estimates of veteran reservists by: (a) age at service exit; (b) sex; (c) Service type; (d) length of service in the UKAF. (TIF)

S2 Table Risk factors for suicide, reserve veterans compared to regular veterans. (PDF)

S3 Table Characteristics of reserve and regular veterans who had contact with mental health services in the 12 months prior to suicide. (PDF)
