# Supplementary material for: Suicide in former reserve UK Armed Forces personnel, 1996–2018: A whole population cohort study
Source: PLOS Ment Health. 2026 Nov 13;3(11):e0000769. doi: 10.1371/journal.pmen.0000769 (PMC13766606; doi:10.1371/journal.pmen.0000769)
Supplement: Supplementary file 1 [file pmen.0000769.s001.pdf]

S1_Table: Multivariable analyses with adjusted hazard ratios of significant risk factors associated with suicide in reserve veterans

Variable	Categories	HR	95% CI	*p*-Value
**Age (years) at service exit**	16-19	Base		
	20-24	1.54	0.95-2.51	0.080
	25-29	1.78	1.05-3.04	0.034
	30-34	2.15	1.21-3.82	0.009
	35-39	2.44	1.27-4.70	0.007
	40-44	1.75	0.79-3.88	0.167
	45-49	2.17	1.00-4.69	0.049
	50-54	1.43	0.47-4.34	0.536
	55+	1.04	0.41-2.67	0.932
**Sex**	Male	Base		
	Female	0.31	0.21-0.47	<0.001
**Service**	Army	Base		
	Navy/Marines	0.78	0.32-1.91	0.584
	RAF	0.26	0.06-1.07	0.062
**Rank**	Non officer rank	Base		
	Officer	0.91	0.57-1.47	0.713
	Non-commissioned officer	0.89	0.72-1.10	0.275
**Length of service (years)**	<1	1.23	0.74-2.03	0.430
	1 to <2	0.96	0.58-1.59	0.872
	2 to <3	0.95	0.57-1.60	0.856
	3 to <4	0.68	0.38-1.22	0.194
	4 to <5	0.97	0.53-1.79	0.935
	5 to <10	0.72	0.45-1.17	0.191
	10 to <15	0.70	0.41-1.20	0.199
	15+	Base		

HR = hazard ratio, CI = confidence interval
