# Supplementary material for: Suicide in former reserve UK Armed Forces personnel, 1996–2018: A whole population cohort study
Source: PLOS Ment Health. 2026 Nov 13;3(11):e0000769. doi: 10.1371/journal.pmen.0000769 (PMC13766606; doi:10.1371/journal.pmen.0000769)
Supplement: Supplementary file 2 [file pmen.0000769.s003.pdf]

S2 Table: Risk factors for suicide, reserve veterans compared to regular veterans

**Variable**	**Categories**	**Reserves (number dying by suicide = 584)**	**Regulars (number dying by suicide = 1,086)**	**OR (95% CI)**	***p*-Value**
**Age (years) at service exit**	16-19	24	287	**0.12 (0.07-0.18)**	**0.000**
	20-24	96	321	**0.47 (0.36-0.61)**	**0.000**
	25-29	69	180	**0.67 (0.49-0.92)**	**0.009**
	30-34	68	93	**1.41 (1.00-1.98)**	**0.042**
	35-39	46	70	1.24 (0.82-1.86)	0.273
	40-44	19	79	**0.43 (0.24-0.72)**	**0.001**
	45+	262	56	**14.97 (10.84-20.95)**	**0.000**
**Gender**	Male	557	1046	0.79 (0.47-1.35)	0.351
	Female	27	40	1.27 (0.74-2.14)	0.351
**Service**	Army	567	799	**11.98 (7.24-21.08)**	**0.000**
	Navy	5	165	**0.05 (0.02-0.12)**	**0.000**
	RAF	12	122	**0.17 (0.08-0.30)**	**0.000**
**Rank**	Non-officer rank	371	761	**0.75 (0.60-0.94)**	**0.009**
	Commissioned Officer	20	41	0.91 (0.50-1.61)	0.730
	NCO	183	271	**1.39 (1.10-1.74)**	**0.004**
**Type of discharge**	Voluntary (normal, own request)	406	325	**4.73 (3.71-6.03)**	**<0.001**
	Involuntary (disciplinary, medical, administrative)	140	530	**0.21 (0.17-0.27)**	**<0.001**
**Deployed on combat operation**	No	556	899	**4.13 (2.72-6.47)**	**0.000**
	Yes	28	187	**0.24 (0.15-0.37)**	**0.000**
**Length of service (years)**	<1	75	328	**0.34 (0.26-0.45)**	**0.000**
	1 to <2	66	98	1.28 (0.91-1.81)	0.136
	2 to <3	52	76	1.30 (0.88-1.90)	0.163
	3 to <4	25	60	0.76 (0.45-1.25)	0.270
	4 to <5	19	71	**0.48 (0.27-0.82)**	**0.005**
	5 to <10	51	203	**0.42 (0.29-0.58)**	**0.000**
	10 to <15	21	65	**0.59 (0.34-0.98)**	**0.035**
	15+	275	185	**4.33 (3.43-5.47)**	**0.000**
