# Supplementary material for: Suicide in former reserve UK Armed Forces personnel, 1996–2018: A whole population cohort study
Source: PLOS Ment Health. 2026 Nov 13;3(11):e0000769. doi: 10.1371/journal.pmen.0000769 (PMC13766606; doi:10.1371/journal.pmen.0000769)
Supplement: Supplementary file 3 [file pmen.0000769.s004.pdf]

S3 Table: Characteristics of reserve and regular veterans who had contact with mental health services in the 12 months prior to suicide

Risk group	Variable	Reserves (n=123)	Regulars (n=273)	OR (95% CI)	p-Value
**Demographic features**	Unmarried	80 (67%)	200	0.57 (0.34-0.96)	0.024
	Unemployed	37 (32%)	156	**0.31 (0.19-0.50)**	**<0.001**
	Long-term sick	18 (16%)	19	**2.49 (1.17-5.23)**	**0.008**
	Living alone	51 (44%)	121	0.84 (0.52-1.33)	0.428
	Homeless	<5	10		
**Service characteristics**	In-patient	11 (9%)	21	1.13 (0.48-2.56)	0.750
	Recent (< 3 months) discharge	24 (21%)	38	1.54 (0.83-2.82)	0.133
	Under CR/HT services	15 (15%)	30	1.28 (0.61-2.59)	0.469
	Missed last contact	30 (28%)	73	0.88 (0.51-1.50)	0.631
	Non-adherent with medication	15 (13%)	29	1.03 (0.49-2.11)	0.921
**Clinical characteristics**	Schizophrenia & other delusional disorders	21 (17%)	41	1.18 (0.63-2.16)	0.580
	Affective disorders (depression or bipolar disorder)	48 (39%)	90	1.32 (0.83-2.10)	0.219
	Alcohol dependence/misuse	12 (10%)	31	0.88 (0.40-1.85)	0.729
	Drug dependence/misuse	<5	24		
	Personality disorder	9 (7%)	34	0.53 (0.22-1.19)	0.105
	Any PTSD diagnosis[b]	<5	9	0.27 (0.01-1.95)	0.239
	Any secondary diagnosis	66 (55%)	154	0.90 (0.57-1.43)	0.661
**Behavioural characteristics**	History of illness < 12 months	30 (27%)	59	1.15 (0.66-1.96)	0.601
	History of self-harm	79 (66%)	173	0.92 (0.57-1.51)	0.740
	History of violence	36 (31%)	77	0.97 (0.58-1.60)	0.903
	History of alcohol misuse	70 (59%)	160	0.89 (0.56-1.43)	0.691
	History of drug misuse	40 (35%)	133	**0.49 (0.30-0.80)**	**0.002**
**Contact with services**	First contact with MHS < 12 months	39 (39%)	89	1.14 (0.68-1.88)	0.640
	Last contact < 7 days	57 (48%)	101	1.50 (0.95-2.38)	0.067
	Long-term risk: low or none	56 (54%)	134	0.77 (0.47-1.28)	0.287
	Short-term risk: low or none	91 (82%)	190	1.08 (0.58-2.04)	0.802
